# In Vitro Laser Treatment Platform Construction with Dental Implant Thread Surface on Bacterial Adhesion for Peri-Implantitis

**DOI:** 10.1155/2017/4732302

**Published:** 2017-07-16

**Authors:** Hsien-Nan Kuo, Hsiang-I Mei, Tung-Kuan Liu, Tse-Ying Liu, Lun-Jou Lo, Chun-Li Lin

**Affiliations:** ^1^Institute of Engineering Science and Technology, National Kaohsiung First University of Science and Technology, Kaohsiung, Taiwan; ^2^Department of Biomedical Engineering, National Yang-Ming University, Taipei, Taiwan; ^3^Department of Surgery, Chang Gung Memorial Hospital, Taoyuan, Taiwan

## Abstract

This study constructs a standard in vitro laser treatment platform with dental implant thread surface on bacterial adhesion for peri-implantitis at different tooth positions. The standard clinical adult tooth jaw model was scanned to construct the digital model with 6 mm bone loss depth on behalf of serious peri-implantitis at the incisor, first premolar, and first molar. A cylindrical suite connected to the implant and each tooth root in the jaw model was designed as one experimental unit set to allow the suite to be replaced for individual bacterial adhesion. The digital peri-implantitis and suite models were exported to fulfill the physical model using ABS material in a 3D printer. A 3 mm diameter specimen implant on bacterial adhesion against* Escherichia coli *was performed for gram-negative bacteria. An Er:YAG laser, working with a chisel type glass tip, was moved from the buccal across the implant thread to the lingual for about 30 seconds per sample to verify the in vitro laser treatment platform. The result showed that the sterilization rate can reach 99.3% and the jaw model was not damaged after laser irradiation testing. This study concluded that using integrated image processing, reverse engineering, CAD system, and a 3D printer to construct a peri-implantitis model replacing the implant on bacterial adhesion and acceptable sterilization rate proved the feasibility of the proposed laser treatment platform.

## 1. Introduction

Dental implants have become a common treatment for missing teeth owing to the advantages in reconstructing the missing tooth form and function without having to prepare the adjacent teeth [1]. The successful dental implant concerns at the early stage are related to osseointegration at the implant-bone interface, while avoiding bone resorption over the long term. The dental implant success rate can reach 90% at the early stage [2]. However, there are still complications such as abutment screw fracture, soft tissue penetration, mucosal inflammation, implant loosening, and bone resorption in long-term treatment [3]. One reason for these complications is peri-implantitis, which is caused by dental calculus attaching to the implant surface leading to periodontal immune reaction, subsequently causing periodontal inflammation and bone loss [4, 5]. The peri-implantitis is usually treated using conservative mechanical debridement, air abrasives, antiseptic treatment, laser, and so on. However, all of these methods have no consistent clinical standard [6]. Muthukuru et al. compared nonsurgical methods such as topical antibiotics, air abrasives, and Er:YAG (Erbium doped: Yttrium-Aluminum-Gamet) laser treatment and found that these three methods can all be remitted for peri-implantitis [6].

The Er:YAG laser was first proposed by Zharikov in 1974 [7]. It is a 2940 nm wave length laser with the closest connection to water among the existing lasers. It can be applied to both hard and soft tissues, cut and treated under water conditions. This dental laser has the highest potential for peri-implantitis treatment. Many studies focused on periodontal diseases suggested that using the Er:YAG laser with the chisel type tip [8, 9] and the angle with tooth surface should be 20°–40° to meet the cleaning dental calculus requirement, while avoiding hurting the cementum. It was also found that laser treatment efficiency is related to parameters such as power, treatment time, tip type, and tooth position. However, a smooth titanium plate is usually used as the test specimen to investigate the parameter influence in laser treatment in most current researches [10, 11]. The smooth titanium plate surface is quite different from the dental implant thread surface and the thread shape on the implant surface may have a covering or reflection effect to the laser light. The laser illumination angle limitation caused by the interference of nearby teeth and surrounding bone resorption can make the clinical situation different from in vitro metal plate experiments and bring doubt to the result. Unfortunately, until now there has been no in vitro laser experiment platform that considers different tooth positions with the levels of periodontal diseases using a real dental implant with threaded surface.

The bacteria description in Er:YAG laser treatment for peri-implantitis in the current literature is relatively scarce. It is now known that periodontal disease or peri-implantitis is caused by gram-negative bacteria and negative lipopolysaccharide body (LPS) produced by anaerobic bacteria [12–14]. Common periodontal bacteria include* Porphyromonas gingivalis*,* Prevotella intermedia*,* Prevotella denticola*; these periodontal bacteria can cause inflammation and immunity changes in periodontal tissues, inducing the osteophage excitation for osseointegration failure and bone resorption [15, 16]. In 2016, Chen et al. used three kinds of intervention-plastic curettage, air-powder abrasive system, and Er:YAG laser to verify the sterilization effect on a titanium surface for dental implant* Escherichia coli *bacteria adhesion [17]. The results showed that the Er:YAG laser can kill bacteria effectively and does not hurt the implant surface.

This study constructed a standard in vitro laser treatment platform that complies with real clinical situations, including different teeth positions and levels of periodontal diseases. This platform permits laser tip treatment at different teeth positions with varying angles. The implant with bacterial adhesion was designed using an extraction suite that can replace the bacterial adhesion implant extraction. Er:YAG laser treatment for peri-implantitis was performed to verify the test platform and sterilization feasibility.

## 2. Materials and Methods Peri-Implantitis Model

**Figure 1:**
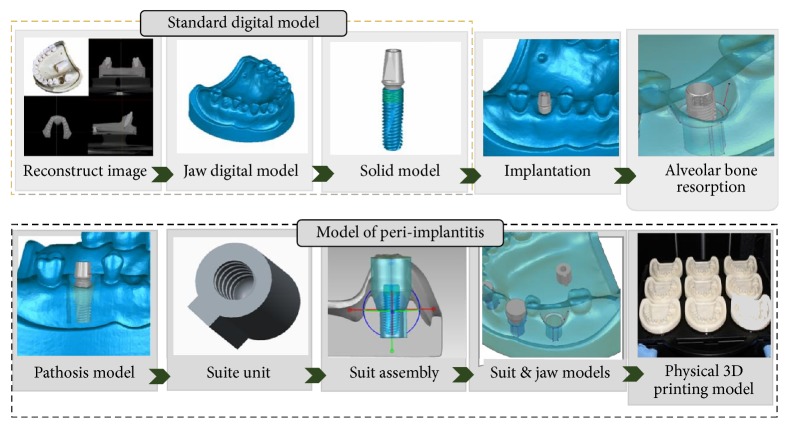
The flowchart for the peri-implantitis in vitro testing model construction.

Figure 1 is the flowchart for the peri-implantitis in vitro testing model construction. The standard clinical adult tooth jaw model (PRO2002-UL-SP-FEM-28, Nissin Dental Products Inc., Japan) and three 11.5 mm length dental implants with diameters of 3 mm, 4 mm, and 5 mm, used for the incisor, premolar, and molar, respectively, were selected as the experimental samples (AnkerSB, Alliance Global Technology Co., Ltd., Taiwan). The i-CAT Dental CT (Imaging Sciences International, PA, USA) were used to scan the tooth jaw model and dental implants. All DICOM CT cross section image data were processed on personal PC using commercially available image-processing software (Mimics® v. 10.01; Materialize Co., Leuven, Belgium) to identify the contours of the different materials. Those contours were extracted and converted into mathematical entities. A 3D digital tooth jaw model and three dental implant solid models were reconstructed in a CAD system (Creo Parametric 2.0, PTC Inc., Needham, MA, USA). Three implants with different diameters assembled in the relative middle incisor, premolar, and molar tooth jaw model positions and implant directions were referenced to the major axis angle of the near teeth.

According to the CIST (Cumulative Interceptive Supportive Therapy) peri-implantitis [18], mechanical debridement and surgical operation classification were needed when the bone loss depth was greater than 5 mm. Following this standard, 6 mm bone loss depth was defined in our model on behalf of serious peri-implantitis, which usually requires a flap in clinical surgery.

The periodontal pocket dimensions were defined as the maximum limitation with the root of the near teeth based on common alveolar bone loss appearance in X-ray images and were as 1.8 mm around the implant for the incisor, 2 mm in the upper part (occlusal direction), and 1.8 mm in the lower part (root direction) for the first premolar and 3 mm in the upper part (occlusal direction) and 2 mm in the lower part (root direction) for the first molar (Table 1). Structural solid models of these three periodontal pockets were constructed and edited in the CAD system.

**Figure 2:**
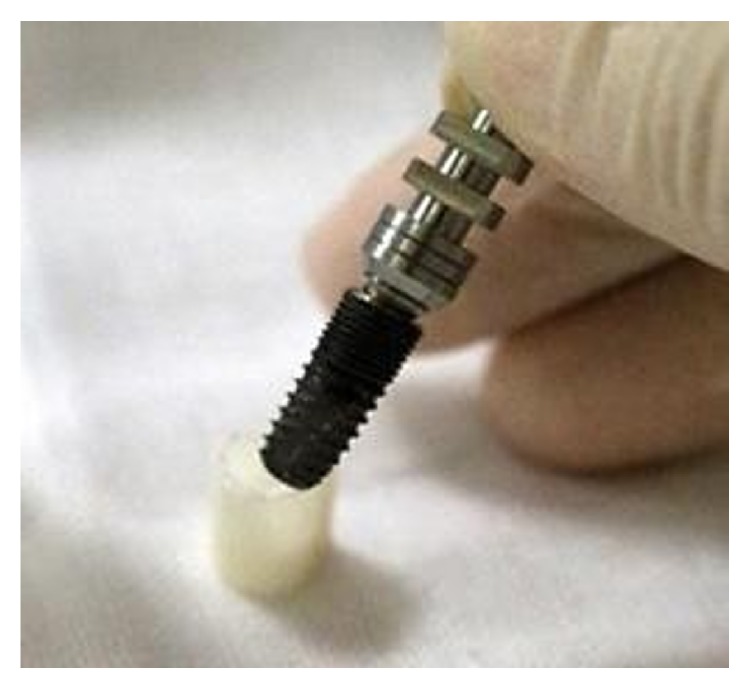
The implant and the suite made one experiment unit set.

A cylindrical suite connected to the implant and each tooth root in the jaw model was designed to permit the suite to be replaced for individual bacterial adhesion. The dental implant and the suite made one experimental unit set in which the bacterial culture can be performed (Figure 2). The digital peri-implantitis and suite models were exported as a stereolithographic (STL) file that can be loaded into a fused deposition modeling (FDM) 3D printer (3DP) with 0.254 mm slicing additive manufacturing (Dimension 1200es SST, Stratasys, Ltd., Minnesota, USA) to duplicate the ABS material (ABS-P430, Stratasys, Ltd., Minnesota, USA).

## 3. Bacterial Culture and Adhesion

**Figure 3:**
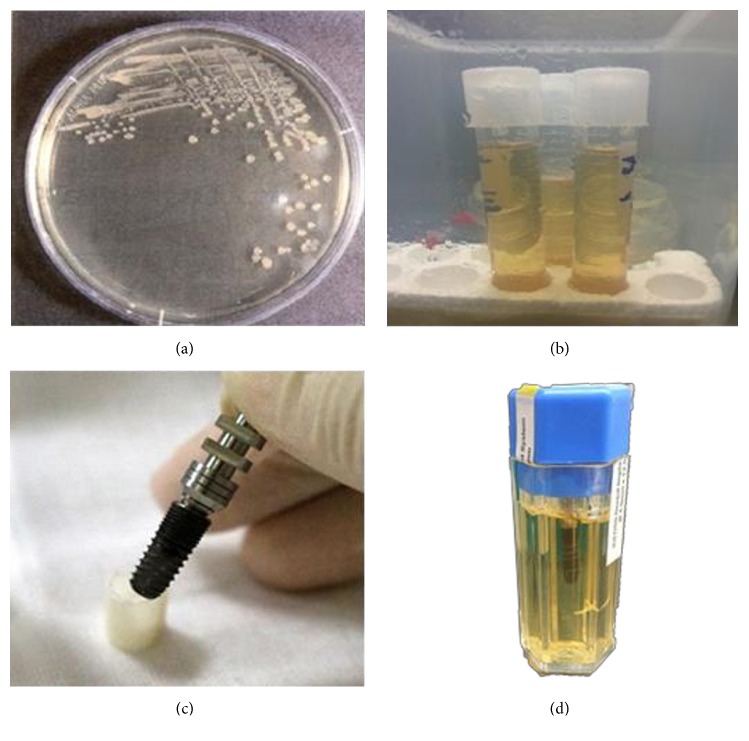
Bacterial culture. (a) Agar culture. (b) Broth culture. (c) Installed implant specimen. (d) Bacteria* (Escherichia coli)* culture with implant.

The jaw model at the incisor was used to verify our platform feasibility in bacterial adhesion and laser sterilization experiments. Three samples were performed in both experiments. The bacterial adhesion to the specimens against* Escherichia coli* (ATCC®25922™ KWIK-STIK, Microbiologics, Inc., USA) was investigated as a model for gram-negative bacteria. Bacteria were cultured onto the solid medium (Agar base, Oxoid Ltd., Basingstoke, Hampshire, UK) and placed into an incubator (37°C) for 24 hours for agar cultivation (Figure 3(a)). Broth medium cultivation was then performed in a spectrophotometer (DU800, Beckman Coulter, Fullerton, CA, USA) operated in the wavelength 600 nm with read average time 0.5 sec (at three circulation) to measure its liquid OD (Optical Density) for quantized correction (Figure 3(b)).

A 3 mm diameter implant and corresponding suite were used to fulfill a set for the bacterial adhesion experiment (Figure 3(c)). The set was placed into medium at 37°C temperature incubator for 24 hours (Figure 3(d)) and 2 ml medium added to centrifuge tubes. The liquid OD was measured with a spectrophotometer and diluted 10 : 1. Plate count was performed to test the adhesion effect after 5 minutes shaking using an ultra-sonicator.

**Figure 4:**
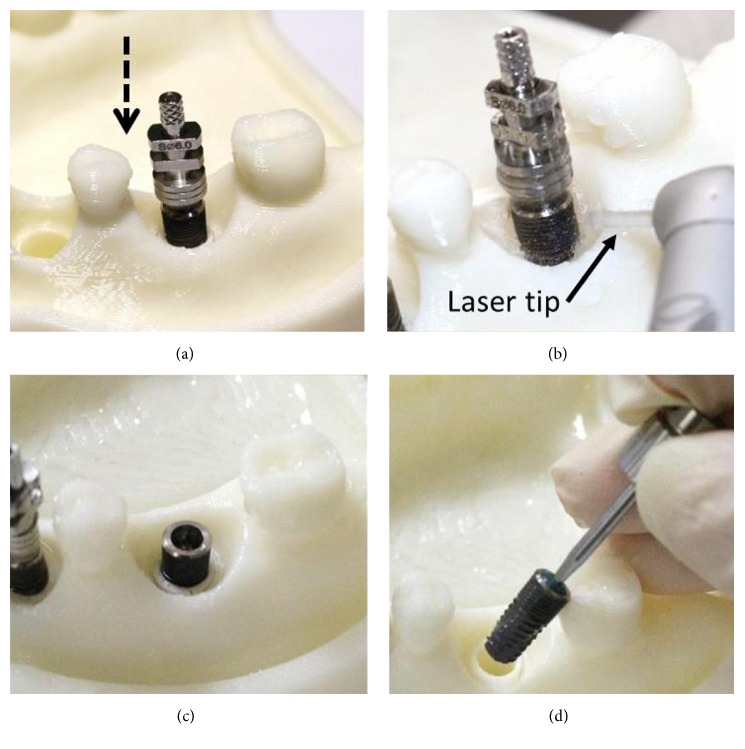
Laser experiment process. (a) Implantation model. (b) Laser treatment. (c) Removal of implant abutment. (d) Removal of implant.

Three dental set samples were assembled into the 3DP jaw model and irradiated with the Er:YAG laser (Er:YAG laser, LightMed Dental Technology Corp., Taiwan) working at 2940 nm with pulse energy at the tip*∗*85 mJ/pulse. A periodontal hand piece was used with a chisel type glass tip (Figure 4). The application tip was moved from the buccal across the implant thread to the lingual and occlusal to the root directions for about 30 seconds per sample. The abutment was then removed and the implant with healing cap was disassembled to form the model set. The implant was placed into a centrifuge tube for the bacteria count (Figures 4(c) and 4(d)). The bacteria number was counted by plate count after 5 minutes shaking using an ultrasonicator (Elmasonic P, Elma Group Inc., Pforzheim, Germany) with frequency 37 kHZ at 21°C for continuity. The *T*-test method was performed to understand the variations between different groups.

## 4. Results

**Figure 5:**
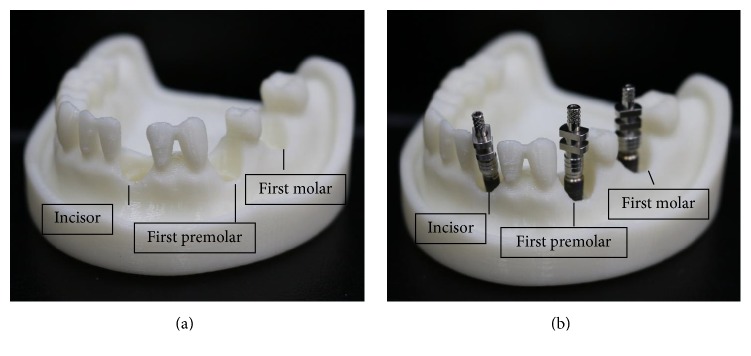
Physical ABS 3D printing model of peri-implantitis for (a) jaw tooth model; (b) jaw tooth and three implants.

Figure 5(a) shows the peri-implantitis in vitro testing model with 3DP jaw ABS model with different teeth positions such as incisor, premolar, and molar. Different dental implants can be assembled and replaced in the 3DP jaw model based on the clinical requirement (Figure 5(b)).

The results indicated that gram-negative bacterium* Escherichia coli *can be cultured to find the calibration curve. The number of bacterial colonies was 10^7^ when the OD value was 0.1. The bacteria adhered onto the implant successfully about 1.01% to 3.83% of the adhesion rate after 24 hours of culture owing to micropores on the implant surface (Table 2). The Er:YAG dental laser sterilization result showed that the sterilization rate can reach 99.3% (standard deviation is ±1.03) (Table 3). Noncontact video measurement system (SVP-2010, ARCS Co., Ltd., Taichung, Taiwan) observations were performed in evaluating jaw model defects. The images were obtained using 37.5 times magnification with a color CCD camera and transferred into an imaging program to evaluate whether the jaw model fractured/melted. No damage was found on the jaw model after laser irradiating testing.

## 5. Discussion

No standard model exists that represents all clinical situations because of the large variation in tooth, dental arch, and alveolar bone anatomy and the different peri-implantitis periodontal pocket structures, bone absorption, degree of inflammation, and implant types found in individual patients. Therefore, many dental laser treatment parameters do not comply with the actual clinical status. The corresponding results from using a smooth disk titanium plate as the jaw model in the literature are quite different from the dental implant thread surface.

An adult tooth jaw model was selected as the standard model due to the shape of its teeth, and arch form and alveolar bone are similar to that in the common healthy adult. This artificial model is also a highly acceptable model for the dentist to practice with and study in the clinic. Although this jaw model was used to simulate the appearance of nearby tooth mesial and distal peri-implantitis and arch form sides, different levels of peri-implantitis at different teeth needed to be defined in detail and created in the standard digital jaw model. The bone loss depth and periodontal pocket were currently used to indicate the severity of peri-implantitis disease; axisymmetric funnel bone loss shape around the tooth was constructed based on X-ray images from clinical patients using reverse engineering and CAD system to mimic the peri-implantitis geometry. The digital peri-implantitis models combined with a novel designed suite can replace implant output to fulfill the final peri-implantitis physical model using ABS material and a 3D printer. The in vitro testing platform construction for peri-implantitis can be performed after bacterial adhesion onto the implant thread surface to irradiate for sterilization.

Although this artificial model cannot reproduce all peri-implantitis states it is relatively real and similar to clinical situations. The mesial/distal influence of nearby teeth and alveolar bone anatomy was considered in the laser system sight and route. A complicated implant thread surface was used instead of the previous smooth disk metal surface to receive radiation vertically. Bone loss depth quantification and different tooth positions can also provide better study parameters for clinical dentists.

Peri-implantitis is caused by gram-negative and anaerobic bacteria. Two major concerns were considered to use* Escherichia coli* as the target bacteria in our sterilization verification study. One was that the objective of this study was to construct a standard in vitro laser treatment platform including different teeth positions placed implant with bacterial adhesion and levels of periodontal diseases. The bacterial culture and adhesion procedures need easy control and stability on the implant. Another concern was peri-implantitis usually caused by gram-negative bacteria and negative lipopolysaccharide body (LPS) produced by anaerobic bacteria [12–14]. Nevertheless,* Escherichia coli *is easier to achieve than that of gram-positive strains with their comparably massive cell-wall-structure [17]. It was validated to adhere onto the implant surface in the literature and the requirement for anaerobic bacterial growth environment is strict and difficult to control [17]. Although the laser methods used by dentists differ due to habit, 98.9% sterilization rate was reached in our peri-implantitis model with a dead angle of nearby mesial/distal teeth under constant laser power. A better in vitro platform was constructed for future laser treatment time research or other parameters which is closer to actual clinical situations.

## 6. Conclusion

This study integrated image processing, reverse engineering, and CAD system to design a peri-implantitis model with different teeth positions in a jaw model. A physical peri-implantitis jaw model made using the ABS 3D printer and individual implant thread surface can be output for bacterial adhesion. The verification experiment used the Er:YAG laser for 30 seconds on the implant surface, reaching 98.9% sterilization rate. These results prove the feasibility of our platform and meet the clinical requirement.

## Figures and Tables

**Table 1:** Implant placed positions and definition of alveolar bone resorption (periodontal pocket dimensions) for incisor, premolar, and molar.

Position	Implant position and alveolar bone resorption			
	Height	Position	Angle	Alveolar resorption
Incisor	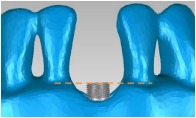	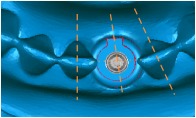	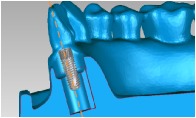	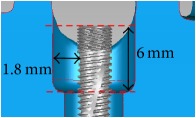

Premolar	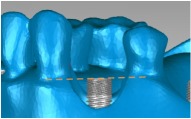	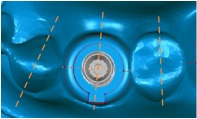	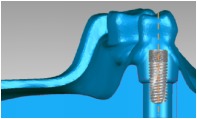	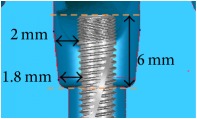

Molar	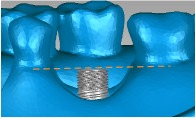	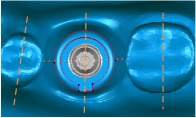	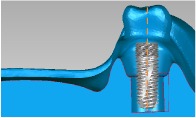	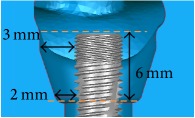

**Table 2:** Dental implants on bacterial adhesion.

	Number of bacteria (*N*_*b*_) (CFU/ml)	Bacterial adhesion (*N*_ba_) (CFU/ml)	Adhesion percentage (*N*_ap_) (%) $N_{ap}=\left[\frac{N_{ba}}{N_{b}}\right]\times 100\%$
1	2.799 × 10^7^	3.5 × 10^5^	1.25
2	2.235 × 10^7^	8.6 × 10^5^	3.83
3	2.388 × 10^7^	2.4 × 10^5^	1.01

**Table 3:** Dental laser sterilization.

	Number of bacteria (*N*_*b*_) (CFU/ml)	Bacterial residual (*N*_br_) (CFU/ml)	Sterilization rate (*N*_sr_) (%) $N_{sr}=\left[\frac{N_{b}-N_{br}}{N_{b}}\right]\times 100\%$	Mean ± SD
1	3.189 × 10^5^	0	100	99.3 ± 1.03
2	2.84 × 10^6^	3.3 × 10^3^	99.8	
3	5.333 × 10^5^	1 × 10^4^	98.1	
